# Reframing risks in rare diseases: economics of networks, spillovers, and scale

**DOI:** 10.3389/fphar.2024.1516725

**Published:** 2024-12-10

**Authors:** Carlisle Ford Runge, James Campbell, Carlisle P. Runge

**Affiliations:** ^1^ Department of Applied Economics, University of Minnesota, St. Paul, MN, United States; ^2^ Division of Health Policy and Management, University of Minnesota School of Public Health, Minneapolis, MN, United States; ^3^ Binera, Inc. Consulting for United States Department of Health and Human Services, Administration for Strategic Preparedness and Response, Biomedical Advanced Research and Development Authority (BARDA), Washington, DC, United States

**Keywords:** rare diseases, economics, networks, spillovers, scale, clustering, drug repurposing, basket trials

## Abstract

Rare diseases affect over three hundred million individuals globally. Investment in research and development remains incommensurate with the challenges rare diseases pose. Further investment in information sharing platforms to promote common and standardized network technologies for rare disease is needed. Rare disease R&D generates information and assets that spill over in other ways, providing benefits that may not be apparent to investors *ex ante.* Analytical and computational methods recently applied at scale are promising. One important way of achieving efficiencies of scale in R&D is clustering rare diseases into groups with similar traits.

## 1 Introduction

Rare diseases affect over three hundred million individuals globally, yet investment in research and development remains incommensurate with the challenges rare diseases pose. In the U.S. only 5 percent of rare diseases have an FDA-approved drug treatment, resulting in an outsized economic burden (Fermaglich and Miller, 2023). Together, these diseases cost the U.S. economy an estimated $1 trillion annually, and in hospital settings cost nearly 90% as much as common conditions (Garrison et al., 2022). This is likely an underestimate due to underreporting and inconsistent coding (Tisdale et al., 2021).

These aggregate impacts represent an underappreciated pool of human health risk and a chance to reframe the investment risk of addressing rare diseases. Economic analysis has an important role to play in better estimates of total costs, rare disease policy, and proposed investment financing structures (Runge et al., 2024). However, the investment proposition of rare diseases remains a challenge (Levine and Stemitsiotis, 2024). The U.S. Orphan Drug Act recognized over 40 years ago that low prevalence diseases have isolated social and economic footprints and therefore merit special attention. This continues to shape the approaches of government and commercial actors at various stages of the R&D pipeline.

With more than 7,000 known rare conditions, it is challenging to scale investment sustainably across this crowded-but-lonely landscape, in which respect for the individuality of rare diseases is important, but strategy and evaluations are needed that look across the rare disease terrain. Recognition of the economic forces connecting rare diseases with one another and with more common diseases is essential to bridge the gap from individual suffering to market-sized solutions. Three related ideas from economics—network effects, positive spillover externalities, and economies of scale—provide a basis for new R&D strategies.

### 1.1 Network effects

Rare disease patient registries, clinical databases, and research networks all involve adoption of information technology. Market demand for these goods and services is subject to network effects, an idea dating to the 1980s in the context of the early internet. A key insight is that the benefit of network participation increases with the number of users (Katz and Shapiro, 1985). Unfortunately, only a subset of rare diseases have digital networks of patients, caregivers, and researchers, with few interoperable ontologies or common datasets linking them together or to more common disorders (Hageman et al., 2023). Naturally, these networks often grow within a single disease community and depend on internal resources and external social awareness, reflecting a particular diseases’ isolation in information-space.

To take advantage of network effects that extend beyond specific disease boundaries, further investment in information sharing platforms to promote common and standardized network technologies for rare disease is needed. This will allow faster, cheaper R&D growth across disparate rare disease efforts, including R&D at all stages of maturity. The Italian neuromuscular diseases registry is one example of a network in which all stakeholders participate with clear roles and responsibilities, favoring patient empowerment, clinical trial design and recruitment, and post-marketing drug surveillance (Ambrosini et al., 2018). On-going efforts such as the Rare-X platform by Global Genes and the IAMRARE platform by NORD provide encouraging starts toward broad, cross-disease networks. As recognition of the need to scale and globalize data sharing on rare diseases increases, network effects provide an additional rationale for such efforts (Nabbout et al., 2023).

Network technologies can be viewed as R&D infrastructure that should be publicly underwritten and shared, such as the rare disease research networks and networks-of-networks in the United States and European Union. Additional public-private collaboration in such networks can help accelerate data exchanges or research marketplaces (Hedley et al., 2023). Investment strategies incorporating network effects also align with life science investors’ growing interest in platforms and supporting technologies.

### 1.2 Positive spillover externalities

In addition to network effects, rare disease R&D generates information and assets that spill over in other ways, providing benefits that may not be apparent to investors *ex ante*. Examples include research knowledge, pharmacological innovations, and new or innovative treatments. These collateral benefits flow from work on one rare disease to another and from rare disease R&D to more common diseases with larger patient populations and market opportunities. Recognizing and accounting for these positive externalities can change our understanding of the social welfare returns to rare disease investment. Investors who internalize and capitalize on these spillovers may improve direct returns on investment and reduce portfolio risks.

It is often difficult to predict and price the ultimate value of scientific and biomedical investigation, but with rare diseases, spillovers may be especially underappreciated. Positive spillovers from research, resulting either from gradual pooling of knowledge or major breakthroughs, imply that research strategies should attempt to recognize rare-to-rare and rare-to-common disease affinities in advance.

One important example of a treatment spillover is drug repurposing. Investors, non-profits, and public agencies increasingly recognize the efficiencies of finding rare disease cures from previously approved or studied therapeutics (Roessler et al., 2021). The nonprofit Every Cure^1^, was recently created at the University of Pennsylvania to build an open-source database for rare disease drug repurposing. Inexpensive generic drugs could ideally be repurposed for rare diseases, but other directions of treatment and research spillovers are also possible, from both rare-to-rare and rare-to-common diseases (Ma et al., 2023). Together, expanding networks and encouraging spillovers can improve the rate of innovation for new cures.

### 1.3 Economies of scale

Researchers and developers operate on the supply-side as producers of knowledge and treatments for rare diseases. Increasing the scale of this production by combining efforts across rare diseases can improve overall efficiency and amplify the impact of R&D. Analytical and computational methods recently applied at scale are promising for patients, clinicians, and investigators collaborating at the industry level. Similarly, within competitive entities, commercial developers are seeking large-scale algorithmic approaches that efficiently identify potential targets and improve the odds of discovery, decreasing risk. Applications to health insurance claims and electronic health records also show promise reducing the diagnostic journey by identifying potentially undiagnosed individuals. Useful insights come from thinking separately about external economies of scale at the industry level and internal economies of scale at the level of the business unit. Considering both external and internal economies of scale can provide an organizing framework.

Because research silos and segmented markets for rare disease treatments discourage sufficient scale, finding and exploiting scaling opportunities is critical. Segmentation occurs in the regulatory and post-approval stages of the therapeutics pipeline as well. Although extended market exclusivity and development subsidies stimulate industry investment in rare disease therapeutics, regulatory regimes generally make approval decisions one candidate at a time, reinforcing isolated market entry and price setting decisions. One important way of achieving efficiencies of scale in R&D is clustering rare diseases into groups with similar traits. Basket clinical trials are an emerging design where eligible patients are grouped by aetiology not disease; these trials have been accepted by regulatory agencies as a basis for drug approvals (Zanello et al., 2023).

### 1.4 Clustering strategies

Clustering rare diseases along different dimensions (organ system, molecular aetiology, or disease agent) can answer both biomedical and pragmatic questions of investment strategy. Clustering rare diseases that affect the lungs or liver simplifies the medical and clinical issues necessary to implement cures, support care, and understand pathogenesis. Angiogenesis disorders, mitochondrial disorders, or rare endocrine malignancies could also be clustered together. The third of rare diseases that are infectious suggest bacterial disease clusters for centralized treatment of infectious agents. In academia and industry, data science approaches are underway to map rare diseases by phenotype, target, and genomic information.

For investors, a pragmatic approach would form clusters with respect to potential biotech acquisitions and strategically combined assets. Deploying capital concentrated in rare disease areas with clustered characteristics, or joining acquisitions such as those amenable to gene therapies, could lower risk and increase expected returns by promoting spillovers within clusters. But large-scale capital investment also requires careful measurement of asset correlation, and diversification to manage risk. Alternative financing structures, such as rare disease drug development megafunds, take advantage of scale and the economics of portfolio theory to motivate innovative R&D investment approaches (Lo and Siah, 2021).

In conclusion, a broad economic perspective of the pipeline for rare disease treatments shows how different stakeholders are working on related issues and how rare and common diseases are linked more often than is recognized. Rare disease investment strategies based on network effects, positive spillover externalities, and economies of scale could result in a more economically healthy biotech environment and faster delivery of cures to patients.

## Data Availability

The original contributions presented in the study are included in the article/supplementary material, further inquiries can be directed to the corresponding author.

## Footnotes

1
https://everycure.org/
